# Electrochemical Detection of Ethanol in Air Using Graphene Oxide Nanosheets Combined with Au-WO_3_

**DOI:** 10.3390/s22093194

**Published:** 2022-04-21

**Authors:** Aynul Sakinah Ahmad Fauzi, Nur Laila Hamidah, Shota Kitamura, Taiga Kodama, Kosuke Sonda, Ghina Kifayah Putri, Takeshi Shinkai, Muhammad Sohail Ahmad, Yusuke Inomata, Armando T. Quitain, Tetsuya Kida

**Affiliations:** 1 Department of Material Science and Applied Chemistry, Graduate School of Science and Technology, Kumamoto University, Kumamoto 860-8555, Japan; aynulsakinahaf@student.usm.my (A.S.A.F.); 207d5214@st.kumamoto-u.ac.jp (S.K.); 210d8811@st.kumamoto-u.ac.jp (T.K.); 212d8815@st.kumamoto-u.ac.jp (K.S.); 212d8829@st.kumamoto-u.ac.jp (G.K.P.); 211d9402@st.kumamoto-u.ac.jp (T.S.); 2 Department of Engineering Physics, Institut Teknologi Sepuluh Nopember (ITS), Surabaya 60111, Indonesia; nurlaila@its.ac.id; 3 Institute of Industrial Nanomaterials, Kumamoto University, Kumamoto 860-8555, Japan; sohail@kumamoto-u.ac.jp; 4 Division of Materials Science, Faculty of Advanced Science and Technology, Kumamoto University, Kumamoto 860-8555, Japan; inomata@kumamoto-u.ac.jp; 5 International Research Organization for Advanced Science and Technology (IROAST), Kumamoto University, Kumamoto 860-8555, Japan; quitain@kumamoto-u.ac.jp; 6 Center for International Education, Kumamoto University, Kumamoto 860-8555, Japan

**Keywords:** graphene oxide, ethanol sensor, proton transport, gold nanoparticles, WO_3_

## Abstract

Detection, monitoring, and analysis of ethanol are important in various fields such as health care, food industries, and safety control. In this study, we report that a solid electrolyte gas sensor based on a proton-conducting membrane is promising for detecting ethanol in air. We focused on graphene oxide (GO) as a new solid electrolyte because it shows a high proton conductivity at room temperature. GO nanosheets are synthesized by oxidation and exfoliation of expanded graphite via the Tour’s method. GO membranes are fabricated by stacking GO nanosheets by vacuum filtration. To detect ethanol, Au-loaded WO_3_ is used as the sensing electrode due to the excellent activity of gold nanoparticles for the catalysis of organic molecules. Au-WO_3_ is coupled with rGO (reduced graphene oxide) to facilitate the electron transport in the electrode. Ce ions are intercalated into the GO membrane to facilitate proton transport. The sensor based on the Ce doped-GO membrane combined with Au-WO_3_/rGO as a sensing electrode shows good electric potential difference (ΔV) responses to ethanol in the air at room temperature. The sensor signal reaches more than 600 mV in response to ethanol at 40 ppm in air, making it possible to detect ethanol at a few ppb (parts per billion) level. The ethanol sensing mechanism was discussed in terms of the mixed-potential theory and catalysis of ethanol on Au-WO_3_.

## 1. Introduction

Detection and analysis of ethanol in human breath are important because of health problems such as breathing troubles, brain cancer, and liver damage. Its safety use is also needed because ethanol leakage and spill sometimes cause fire accidents. Therefore, portable, compact, and small sensors capable of detecting ethanol in the human breath as well as in the atmosphere are required to prevent ethanol-associated health issues and accidents. Besides, ethanol monitoring using portable gas sensors is valuable in the chemical and food industries and the renewable energy sector. SnO_2_-based chemoresistive sensors are often used to detect ethanol [1,2]. However, these type of sensors has a high energy consumption due to the high-temperature operation. Thus, it is crucial to develop a highly sensitive and selective ethanol sensor with a compact structure and low energy consumption.

A solid electrolyte-based mixed-potential-type sensor is one of the promising devices to detect flammable gases such as hydrogen, CO, and hydrocarbons [3,4,5]. This type of device refers to the mixed potential as the sensor signal, which is generated when electrochemical cathodic and anodic reactions occur simultaneously at the triple phase boundary (TPB) between the sensing electrode and the solid electrolyte. The sensor response is measured under open-circuit conditions, where the rates of cathodic and anodic reactions are equal. The sensor response changes with variations in the concentration of combustible gases, following a semi-logarithm relationship between the sensor signal and gas concentration. It has been reported that mixed-potential-type sensors using metal oxides also give a high response and sensitivity to VOCs such as ethanol [6,7,8]. However, most of the solid electrolyte sensors operate at high temperatures of more than 300 °C because the solid electrolytes used in those devices have a low ionic conductivity at lower temperatures. The high-temperature operation increases the power consumption and thus weakens the sensor stability [9]. Furthermore, most of these sensors have a high detection limit, low sensitivity and selectivity, and insufficient stability. Therefore, further materials development is highly demanded.

To overcome those limitations, we focused on graphene oxide (GO) as a base material for a mixed-potential-type gas sensor. GO contains abundant oxygen functional groups at the edge and basal plane of its graphene nanosheet. Because of their presence, GO shows high proton transporting properties; its proton conductivity is as high as that of Nafion at room temperature [10,11]. We have reported that a self-standing membrane composed of GO nanosheets serves as a solid electrolyte for mixed-potential-type gas sensors when fitted with active electrocatalysts [12,13]. However, a membrane made with pristine GO has low thermal and chemical stability. A noticeable decrease in the proton conductivity even at around 60 °C was seen, which is a serious problem. On the other hand, recent reports have proved that adding cations to GO membranes enhances the proton conductivity as well as the stability [14,15]. In this study, we intercalate cerium ions to GO membranes and examine their stability, proton conductivity, and applicability for mixed-potential-type gas sensors.

In addition to the stability, the sensitivity of the GO-based sensor should also be improved for practical uses. There are two main factors to obtain a high sensitivity for a mixed-potential-type gas sensor. The first factor is the electrocatalytic activity of the sensing electrode, which should boost electrochemical reactions at the TPB. The second one is the electrode micro-structure to let target gases easily diffuse to the TPB [4,7]. To achieve efficient diffusion of target gases, the thickness should be adequately controlled. Otherwise, heterogeneous combustion of target gases in the sensing electrode blocks the gas diffusion to the TPB. In this study, we used WO_3_ as the sensing material because it shows an excellent response and sensitivity to various gases such as NH_3_, C_2_H_2_, and NO_2_ when coupled with rGO (reduced graphene oxide) [16,17]. Coupling WO_3_ with rGO would facilitate the electron transport in the electrode. It is also known that doping noble metals such as gold, palladium, and silver on WO_3_ improves the sensitivity [18,19]. Among them, gold has been reported to be catalytically active for the oxidation of organic gases when supported on metal oxides [20]. It is expected that decorating the WO_3_ surface with gold lowers the optimal working temperature of a sensor.

Here, we demonstrate that the mixed-potential-type gas sensor using a Ce-intercalated GO membrane fitted with an Au-WO_3_/rGO electrode is promising for the detection of ethanol at a low ppb (parts per billion) level in air at room temperature.

## 2. Materials and Methods

### 2.1. Fabrication of GO Membranes

A GO suspension was synthesized from expanded graphite (EC1500 and EC500, Ito Graphite Co., Ltd., Kuwana, Japan, mean size: 7.11 and 26.29 µm) via the modified Tour’s method [15]. The effective use of expanded graphite for mass production of GO has been reported by Sun and Fugetsu [21]. Indeed, using expanded graphite in the Tour’s method made the synthesis of GO much easier. Expanded graphite (3.0 g) and potassium permanganate (18.0 g) were mixed in distilled water, followed by the addition of 360 mL of sulfuric acid (98%, Wako Pure Chemical Industries, Osaka, Japan) and 40 mL of phosphoric acid (85%, Wako Pure Chemical Industries). The mixture was then heated at 50 °C for 12 h. Afterward, 400 g of ice, which was made from distilled water, was added to the mixed solution together with hydrogen peroxide until the solution color changed from brown to yellow. The oxidized graphite was then recovered by centrifugation (4000 rpm) for 10 min. An HCl solution (5%, Wako Pure Chemical Industries) was used to remove impurities such as metal ions. The product was further purified by centrifugation in distilled water for 30 min several times. The purified graphite oxide suspended in water was exfoliated by 5 h ultrasonication. The resulting GO nanosheets were recovered using 30 min centrifugation (10,000 rpm). The separated and purified GO nanosheets were kept in distilled water as a suspension (4 mg·mL^−1^) for further use. A Ce ion-intercalated GO membrane was fabricated by vacuum filtration using a membrane filter and 5 mL of a GO suspension containing 0.005 mmol of cerium (IV) sulfate tetrahydrate (Ce(SO_4_)_2_·4H_2_O).

### 2.2. Synthesis of Au-WO_3_ and rGO for the Sensing Electrode

Gold-loaded tungsten trioxide (Au-WO_3_) was synthesized as the electrocatalyst for ethanol detection. Au-WO_3_ was mixed with reduced graphene oxide (rGO) to improve the electron conductivity. 13.5 g of sodium tungstate dihydrate (Na_2_WO_4_·2H_2_O) (purity 85%, Wako Pure Chemical Industries), 2.25 g of sodium dodecyl sulfate (NaC_12_H_25_SO_4_) (purity 95%, Wako Pure Chemical Industries), and 2.25 g of sodium chloride (NaCl) (purity 99.5%, Wako Pure Chemical Industries) were added to 300 ml of distilled water containing synthesized GO in distilled water, and the mixture was stirred for about 1 h. HCl (5%) was added to adjust the pH of the solution to 1.5 under stirring for another 3 h. The mixture was transferred into a Teflon-line stainless steel autoclave and was heated at 180 °C for 24 h. After the reaction, the autoclave was cooled with water. The product was washed with distilled water and methanol by centrifugation at 5000 rpm, dried at 50 °C for 12 h, and annealed in nitrogen at 350 °C for 6 h to produce WO_3_/rGO. For Au loading, a urea-assisted gold deposition method was used [22]. The synthesized WO_3_/rGO (1.0 g) was added to 100 mL of distilled water containing 0.17 g of chloroauric acid (HAuCl_4_) (purity 99%, Wako Pure Chemical Industries) and 2.52 g of urea (CO(NH_2_)_2_) (purity 99%, Wako Pure Chemical Industries), and the solution was stirred at 80 °C for 16 h. The product was washed with distilled water by centrifugation four times and dried at 100 °C for 2 h to obtain Au-WO_3_/rGO.

### 2.3. Materials Characterization

The crystal structure of the synthesized materials was characterized by X-ray diffraction (XRD) (MiniFlex600; Rigaku, Tokyo, Japan) using Cu-Kα radiation (λ = 0.15418 nm). The interlayer space of the GO membrane was also determined by XRD. Fourier transform infrared (FT-IR) spectroscopy (FTIR4100; JASCO, Tokyo, Japan) was performed to analyze the presence of oxygen functional groups on GO. Raman spectroscopy (NRS-3100; JASCO, Tokyo, Japan) was applied to obtain the structural information of the electrode materials. X-ray photoelectron spectroscopy (XPS, PHI1600; PerkinElmer, Waltham, MA, USA) was used to determine the surface state and composition of Au-WO_3_/rGO. Its microstructure was also analyzed by a high-resolution transmission electron microscope (HR-TEM) (Tecnai F20; Thermo Fisher Scientific, Waltham, MA, USA). Electrochemical impedance spectroscopy (EIS, 1260; Solartron, Bognor Regis, UK) was used to measure the proton conductivity of the GO membrane in the frequency range of 1 MHz to 1 Hz at 25 to 100 °C. Brunauer-Emmet-Teller (BET, BELSORP-mini II; Microtrack Bell, Osaka, Japan) analysis was carried out to determine the surface area and total pore volume of the sensing electrode. Thermogravimetry-differential thermal analysis (TG-DTA, SII Exstar 6000; SEIKO, Tokyo, Japan) was carried out to determine the thermal stability of the sensing electrode. The surface morphologies and thickness of the electrodes of the sensor device were examined using scanning electron microscopy (SEM, JSM-7600F; JEOL, Tokyo, Japan).

### 2.4. Sensor Fabrication and Sensing Tests

**Figure 1:**
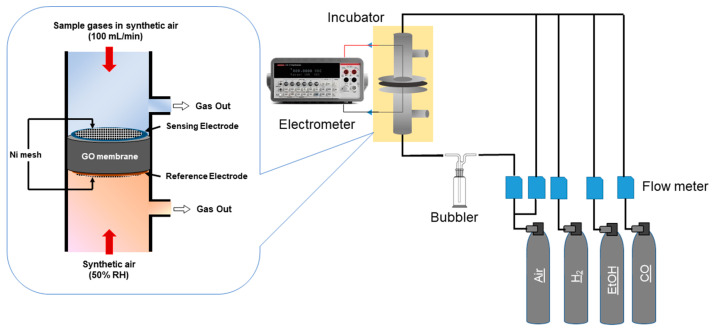
Schematics of the device structure and experimental setup for gas sensing measurements.

Figure 1 shows the schematic diagram of the sensor device and the experimental setup. The device was fabricated in the following manner. Au-WO_3_/rGO was deposited on one side of the GO membrane as the sensing electrode, while Pt(46.3%)/C (TEC10E50E; Tanaka Holdings Co., Ltd., Tokyo, Japan) was applied on the other side as the counter electrode. For the electrode formation, a powder of Au-WO_3_/rGO was dispersed in isopropanol and mixed with a GO suspension to form a paste, which was coated on the membrane. In this case, GO is expected to serve as an ionomer that assists in electrochemical reactions at the TPB. The counter electrode was formed by the same method as noted above. The thicknesses of the sensing and counter electrodes were 177.1 and 500 mm, respectively. The electrodes were covered by Ni meshes with Ni wires (Nilaco Corporation, Tokyo, Japan) on both the sensing and reference sides. The electric potential difference (ΔV) between the sensing and counter electrodes was measured by an electrometer.

Ethanol gases with different concentrations were prepared by blending a commercial ethanol gas (50 ppm in air) with synthetic air. Commercial gases were supplied from Sumitomo Seika Chemicals Co., Ltd., Osaka, Japan. The sample gases were introduced to the sensing electrode side, while synthetic air was introduced to the reference electrode side as a reference gas. We let the reference gas pass through a bubbler containing distilled water to humidify GO membranes because GO loses its proton conductivity in a dry atmosphere. For the sensing side, sample gases were mixed with humidified synthetic air (100% relative humidity (RH)) to control the humidity, which was measured by a humidity sensor (Thermo Recorder TR-72wf, T&D, Matsumoto, Japan). The flow rate of sample gases was set to 100 mL·min^−1^, while that of the reference gas was 20 mL·min^−1^.

### 2.5. Study on Adsorption, Desorption, and Reaction of Ethanol on Au-WO_3_

The temperature-programmed desorption (TPD) of ethanol on Au-WO_3_ were carried out by an automated catalyst analyzer (BELCAT II, MicrotracBEL, Osaka, Japan) connected to a mass spectrometer (BELMass, MicrotracBEL, Osaka, Japan). Before TPD experiments, samples were treated with 20% O_2_ in He for 30 min at 300 °C, followed by purging with He for 15 min. The adsorption of ethanol was performed by introducing 50 ppm ethanol in synthetic air to a chamber where samples were loaded for 30 min at room temperature. After purging the system with He, the samples were heated to 300 °C with a heating rate of 10 °C·min^−1^ in He. Gas molecules desorbed from the samples were monitored by the mass spectrometer.

## 3. Results and Discussion

### 3.1. Materials Characterization

**Figure 2:**
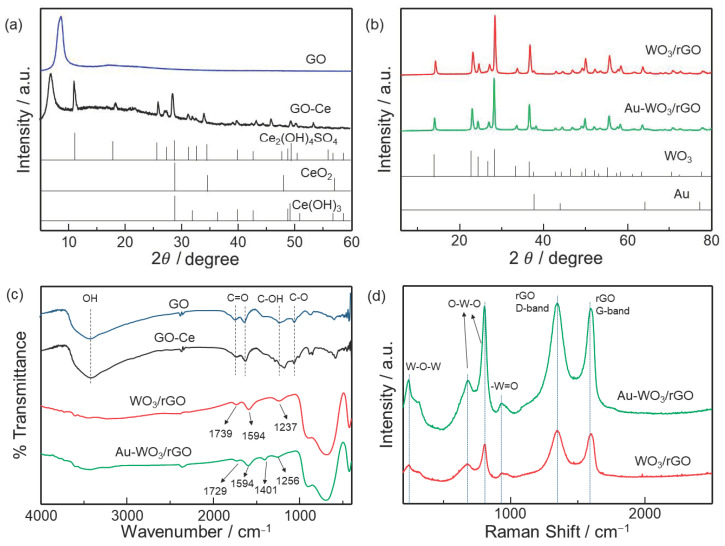
XRD patterns of (**a**) GO, GO-Ce membranes, (**b**) Au-WO_3_/rGO, and WO_3_/rGO. (**c**) FT-IR spectra of GO, GO-Ce membranes, WO_3_/rGO, and Au-WO_3_/rGO. (**d**) Raman Spectra of WO_3_/rGO and Au-WO_3_/rGO.

The interlayer distance of GO membranes with and without Ce was analyzed by XRD analysis, as shown in Figure 2a. A sharp diffraction peak at 2θ = 8.17° was observed for the pristine GO membrane, showing that the interlayer distance of the GO membrane is 1.08 nm. It can be clearly seen that for the GO membrane with Ce ions, the peak shifted to 6.73°, which corresponds to the interlayer distance of 1.31 nm. This is a clear indication that Ce ions were successfully intercalated to the GO membrane. Ce ions should be present in the interlayer of the membrane because of the negative charge of GO nanosheets. Ce ions are also likely to present between GO nanosheets via chelate formation with carboxy groups at the edge of GO nanosheets [23]. In the XRD pattern, other peaks ascribable to CeOSO_4_·H_2_O, CeO_2_, and Ce(OH)_3_ were seen, suggesting the formation of such particles [24,25,26]. The incorporation of Ce-based nanoparticles in the GO membrane might also contribute to the observed increase in the interlayer distance. The Ce-GO membrane showed much better stability in water than the pristine GO membrane. This is because Ce ions suppressed GO nanosheets from dissolving in water via the electrostatic interaction and the chelate formation noted above.

The XRD patterns of as-synthesized WO_3_/rGO powder before and after Au deposition are shown in Figure 2b, which clearly shows the formation of the monoclinic WO_3_ phase (JCPDF 43-1035) with characteristic peaks at 13.9, 28.2, and 37°. On the other hand, the characteristic peak of GO at around 10° disappeared, indicating that GO nanosheets were reduced to rGO by the hydrothermal processing at 180 °C for 24 h. One small peak ascribable to gold was detected at around 38° after Au deposition, indicating that gold was successfully loaded over WO_3_/rGO.

Figure 2c shows FT-IR spectra of as-synthesized GO membranes with and without Ce and WO_3_/rGO before and after Au loading. The presence of hydroxyl, carboxyl, carbonyl, and epoxy groups were confirmed for the GO and GO-Ce membranes. The C-OH peak at 1237 cm^−1^ shifted by the Ce addition, suggesting the interaction of Ce ions with hydroxyl groups of GO. For WO_3_/rGO with and without Au loading, the peak intensities at 3500, 1737, 1640, 1220, and 1058 cm^−1^ ascribable to GO nanosheets were decreased, indicating that GO was reduced to rGO. Broad peaks seen at less than 1000 cm^−1^ refer to the vibration of the W-O-W bond [27,28]. The peak at 1401 cm^−1^ can be ascribed to NH^4+^ ions originating from urea that was used for gold deposition [22].

The structure of WO_3_/rGO with and without gold was examined by Raman spectroscopy, as shown in Figure 2d. The peak at 244 cm^−1^ is the bending mode of the W-O-W bond. The peaks at 680 and 810 cm^−1^ correspond to the asymmetric and symmetric vibrations by the O-W-O stretching modes. The peak at 935 cm^−1^ is the symmetric stretching mode of W=O bond. The presence of all these peaks indicates the formation of the WO_3_ phase. The peaks at 1347 and 1600 cm^−1^ belong to the D and G bands of rGO [27,29,30]. Overall, the Raman peak intensities were significantly enhanced after Au loading. This should be due to the surface-enhanced Raman scattering (SERS) effect, which is clear evidence of the presence of gold nanoparticles on WO_3_/rGO [31,32]. As revealed by TG-DTA (Figure S1 in Supporting Information), the decomposition temperature of the remaining surface functional groups on rGO shifted from 580 to 620 °C. This is another evidence of the presence of catalytically active Au nanoparticles that promotes the catalysis of organic molecules.

**Figure 3:**
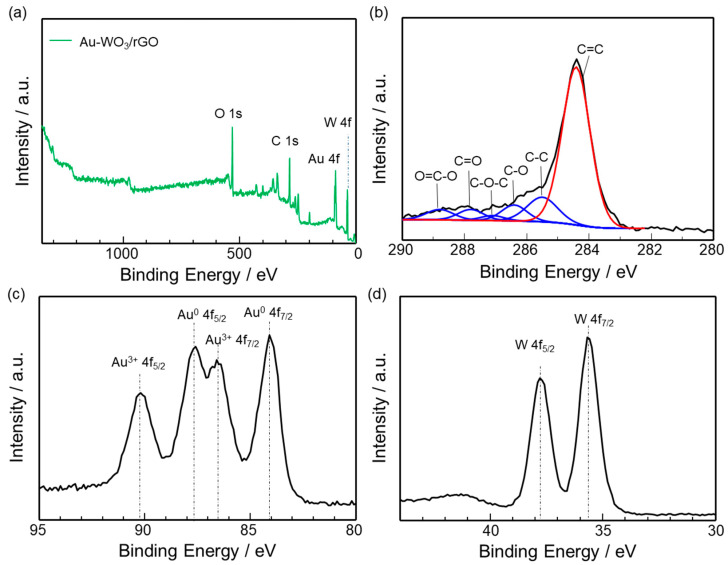
XPS spectra of as-synthesized Au- WO_3_/rGO: (**a**) Survey scan spectrum and high-resolution spectra of (**b**) C 1s, (**c**) Au 4f, and (**d**) W 4f elements.

The chemical bonding state of Au-WO_3_/rGO was examined by XPS. Figure 3a shows the survey spectrum of Au-WO_3_/rGO powder, displaying the presence of elements C, O, W and Au. The atomic ratio of Au: W: C is calculated to be 1.0: 1.2: 12.8. The high-resolution C 1s spectrum (Figure 3b) indicates the presence of C=C, C-C, C-OH, C-O-C, C=O, and O=C-O, which correspond to the observed peaks at 284.4, 285.5, 286.4, 287.1, 287.8, and 288.8 eV, respectively. The reduction of GO produced the most intense peak for C=C. In the Au 4f spectrum (Figure 3c), four peaks were seen at the binding energy from 82 to 92 eV, suggesting the presence of metallic Au^0^ and Au^3+^ species [33,34]. Several papers report the stabilization of Au^+3^ through its interaction with supports [35]. It might be possible that the interaction of gold atoms with oxygen functional groups on rGO stabilized Au^+3^. However, further investigation is necessary to clarify the mechanism. Figure 3d shows the high-resolution W 4f spectrum of Au-WO_3_/rGO. The peaks at 37.8 and 35.7 eV were ascribed to W 4f5/2 and 4f7/2 in WO_3_, respectively, suggesting that the valence state of W is 6^+^ [36].

**Figure 4:**
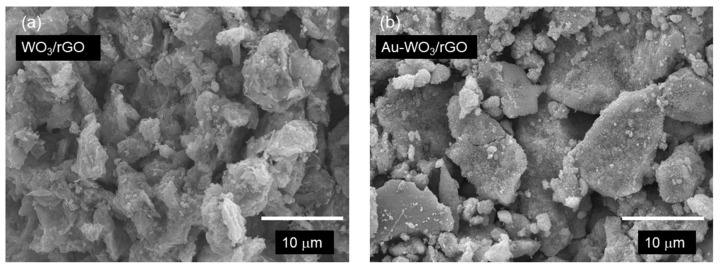
SEM images of (**a**) WO_3_/rGO and (**b**) Au-WO_3_/rGO.

**Figure 5:**
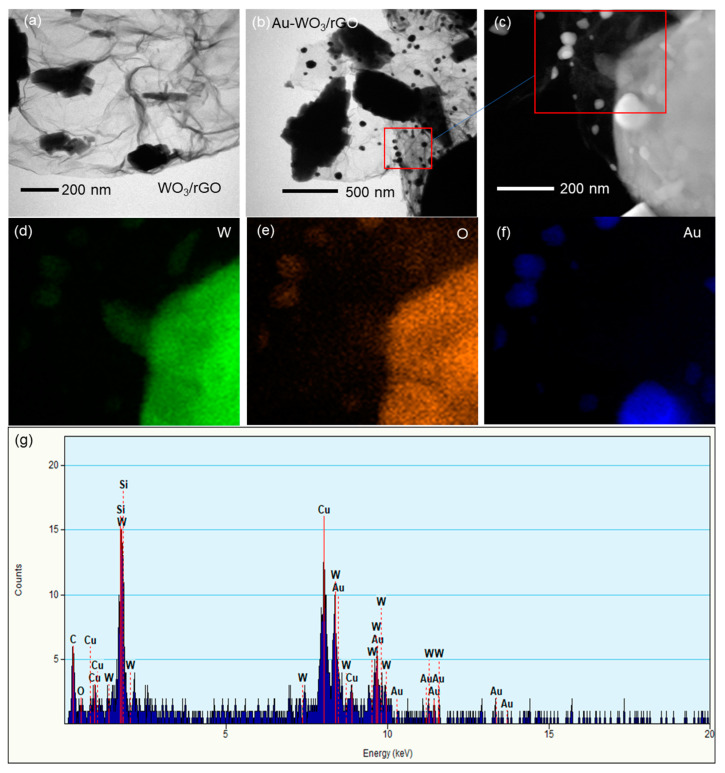
TEM images of (**a**) WO_3_/rGO and (**b**) Au-WO_3_/rGO. (**c**) HAADAF-TEM image of Au-WO_3_/rGO. (**d**–**f**) EDX elemental mapping and (**g**) EDX spot spectrum of Au-WO_3_/rGO. The area marked with the red box in (**b**) is magnified and analyzed by EDX.

The morphology of WO_3_/rGO with and without Au was studied by SEM and TEM. The images in Figure 4a,b show the presence of large rectangle-shaped particles of 6–14 µm in size for both electrodes. Small particles appeared on the large particles after Au loading. TEM analysis (Figure 5a,b) indicates that the large particles were wrapped by wrinkled nanosheets of rGO. In addition, nanoparticles were clearly seen on rGO nanosheets after Au loading. High-angle annular dark-field scanning transmission electron microscopy (HAADF-TEM) and energy-dispersive X-ray (EDX) mapping analyses (Figure 5c–f) revealed that large and small Au particles were observed over WO_3_/rGO. The average specific surface areas of WO_3_/rGO before and after Au loading were 2.38 and 2.22 m^2^·g^−1^, respectively (Table S1). A slight decrease in surface area was observed after Au loading. In addition, the pore volume in the range of 20 to 80 nm decreased after Au loading (Figure S2). Large Au particles of 20–80 nm were probably embedded in the mesopores of WO_3_/rGO.

**Figure 6:**
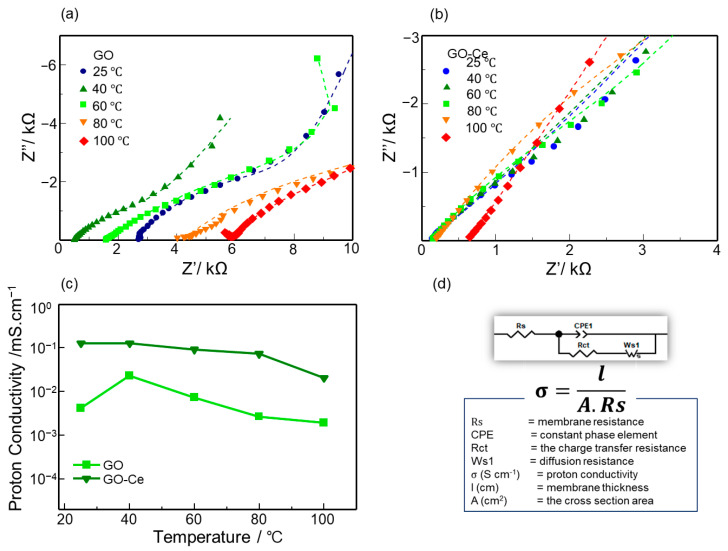
Nyquist plots of (**a**) GO and (**b**) GO-Ce membranes. (**c**) Dependence of proton conductivity on temperature. (**d**) Equivalent circuit of GO membranes and parameters for the curve fitting and conductivity calculation.

To confirm the applicability of the GO membrane for electrochemical applications, its proton conductivity was measured by EIS in the frequency range of 1 MHz to 1 Hz at 25 to 100 °C. Figure 6a,b show the Nyquist plots of the GO and GO-Ce membranes, respectively. The membrane’s bulk resistance (Rs) is represented by the intersection of the curves at the X-axis (real part of impedance) at higher frequencies according to the equivalent circuit shown in Figure 6d, which was used for data fitting. Using Rs values at different temperatures, the bulk proton conductivity of the membranes was calculated. Rs values changed with temperature. Figure 6c shows the dependence of the proton conductivity as a function of temperature for the GO membranes with and without Ce. In the temperature range examined, the proton conductivity of the membrane with Ce was higher than that without Ce. In addition, the conductivity at room temperature was higher than that for Nafion (ca. 10^−2^ S·cm^−1^), indicating its feasibility for use in electrochemical devices [37,38]. However, the proton conductivity decreased as temperature increased, suggesting the desorption of oxygen functional groups. Nevertheless, the improvement in the conductivity should be due to the increase in the interlayer of the membrane by Ce intercalation, which facilitates the diffusion of protons in the interlayer [13]. Further study is in progress to clarify the effect of cations on the proton conductivity of GO membranes.

### 3.2. Ethanol Sensing Properties

**Figure 7:**
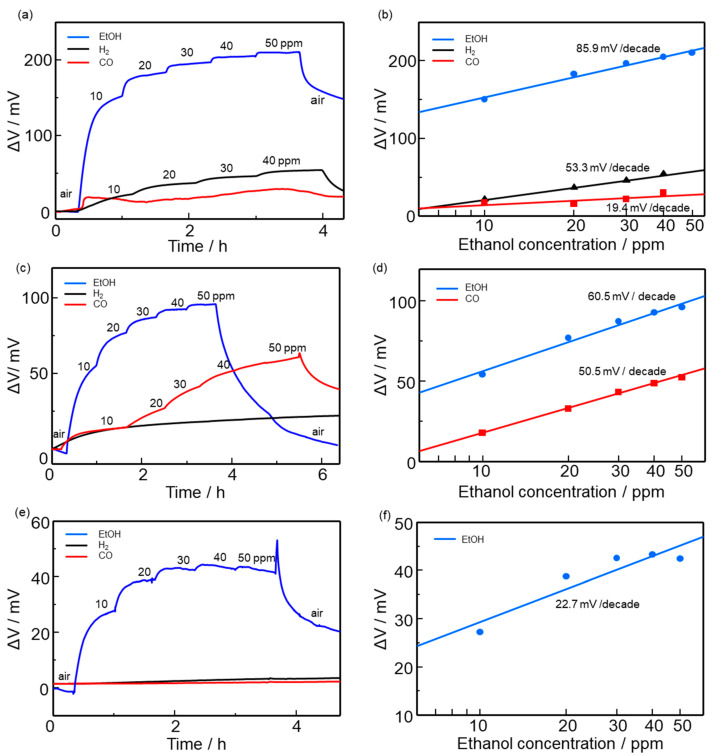
Time dependence of the sensor response (ΔV) for different ethanol concentrations at (**a**) 25, (**b**) 40, and (**c**) 60 °C. Dependence of the sensor response on ethanol concentration at each temperature at (**d**) 25, (**e**) 40, and (**f**) 60 °C.

Figure 7a–c depicts ΔV responses of the device using the GO-Ce membrane fitted with the Au-WO_3_/rGO sensing electrode to ethanol, H_2_, and CO (10–40 ppm) at different temperatures such as 25, 40, and 60 °C. The sensor responded to changes in ethanol concentration in air at all temperatures. Here, the sensor response, denoted as ΔV, was defined as the difference between ΔV in air and air containing combustible gases. The sensor demonstrated a good selectivity to ethanol over hydrogen. The 90% response time at 25 °C was approximately 12 min. The slow recovery speed of the sensor is likely due to the slow desorption of by-products formed by ethanol combustion. Figure 7d–f shows the dependence of ΔV on ethanol concentration at each operating temperature. The sensor response was the highest at 25 °C; the slope of the curve for ΔV vs. concentration was 85.9 mV/decade. This high sensitivity to ethanol allows for the detection of ethanol at a few ppb (parts per billion) level. However, the sensor response decreased as the operating temperature increased.

The gas sensing phenomenon can be explained by the mixed potential mechanism [4,5]. According to the theory, cathodic and anodic reactions occur simultaneously at the TPB of the sensing electrode and the rates of the two reactions are equal under open-circuit conditions. The following reactions are suggested to occur at the sensing electrode composed of Au-WO_3_/rGO to generate the mixed-potential;

(1) $$C_{2}H_{5}OH+\frac{3}{2}O_{2}\to 2CO_{2}+6H^{+}+6e^{-}$$

(or C_2_H_5_OH → CH_3_CHO + 2H+ + 2e^−^) (2)

(3) $$\frac{1}{2}O_{2}+2H^{+}+2e^{-}\to H_{2}O$$

Electrochemical oxidation of ethanol usually proceeds via the formation of incomplete-oxidation products such as acetaldehyde and acetic acid [39]. On the other hand, the equilibrium reaction associated with O_2_ (Equation (4)) may take place at the interface between the Pt-C counter electrode and the GO-Ce membrane. In air (constant oxygen partial pressure), the potential at the counter electrode is fixed, while the potential at the sensing electrode changes with variations in ethanol concentration in air. Thereby, a sensor signal (ΔV) is generated between the sensing and counter electrodes, depending on ethanol concentration [40].

(4) $$\frac{1}{2}O_{2}+2H^{+}+2e^{-}⇌H_{2}O$$

The rate of the anodic reaction determines the mixed-potential as well as the response speed. Thus, the electrocatalytic activity of the sensing electrode plays a key role. The high catalytic activity of Au-WO_3_/rGO for oxidation of organic compounds should be responsible for the high sensitivity and selectivity to ethanol. In addition, the microstructure of the sensing electrode is important to achieving a high reaction rate at the TPB. To promote the anodic reaction at the TPB, we controlled the thickness of the sensing electrode.

**Figure 8:**
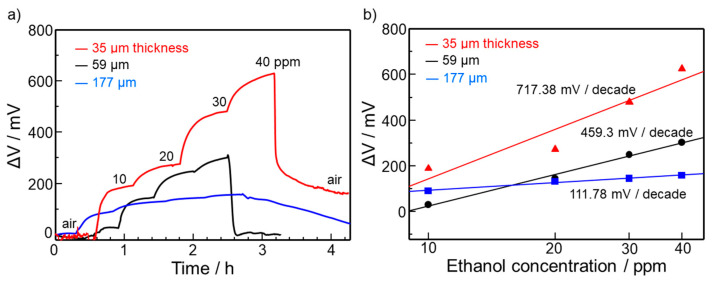
(**a**) ΔV responses to ethanol at 25 °C for GO-Ce devices using the Au-WO_3_/rGO sensing electrodes with different thicknesses. (**b**) Dependence of ΔV on ethanol concentration for the devices with different electrode thicknesses.

Figure 8a,b shows the sensor response of devices to ethanol (10 to 40 ppm in air) at 25 °C using sensing electrodes with different thicknesses (34.7, 58.7, and 177.1 µm). The sensor response increased as the thickness decreased. In addition, the response speed was also improved by decreasing the thickness. Accordingly, the slope of the sensor response curve on ethanol concentration significantly increased from 111.78 to 717.38 mV/decade, making ultrasensitive detection of ethanol possible. The reason for the enhancement in sensor response can be explained in terms of the gas diffusion effect. Decreasing the electrode thickness facilitates ethanol diffusion deep inside the electrode. Thus, the number of ethanol molecules that reach the TPB increased, thereby generating a large change in sensor response. In contrast, for the thick electrode, the ethanol diffusion is retarded by the heterogeneous catalytic combustion of ethanol in the sensing electrode. The observed decrease in the sensor response at higher temperatures seen in Figure 7 can also be explained in terms of the diffusion effect. The ethanol diffusion to the TPB is decelerated by the heterogeneous catalytic combustion that is activated at higher temperatures. Note that the recovery of the response became slow for the thin electrode (34.7 µm). This is probably because of the accumulation of by-products that were formed by the incomplete combustion of ethanol at the TPB. Thus, the control of the microstructure, such as the pore size of the sensing electrode, should be carried out to assist in the desorption of by-products from the TPB. In this study, sensing measurements were carried out in dry conditions, although the reference electrode was humidified. It has been reported that the proton conductivity of GO depends on humidity [10]. Thus, it is possible that the sensor response is also influenced by humidity.

**Figure 9:**
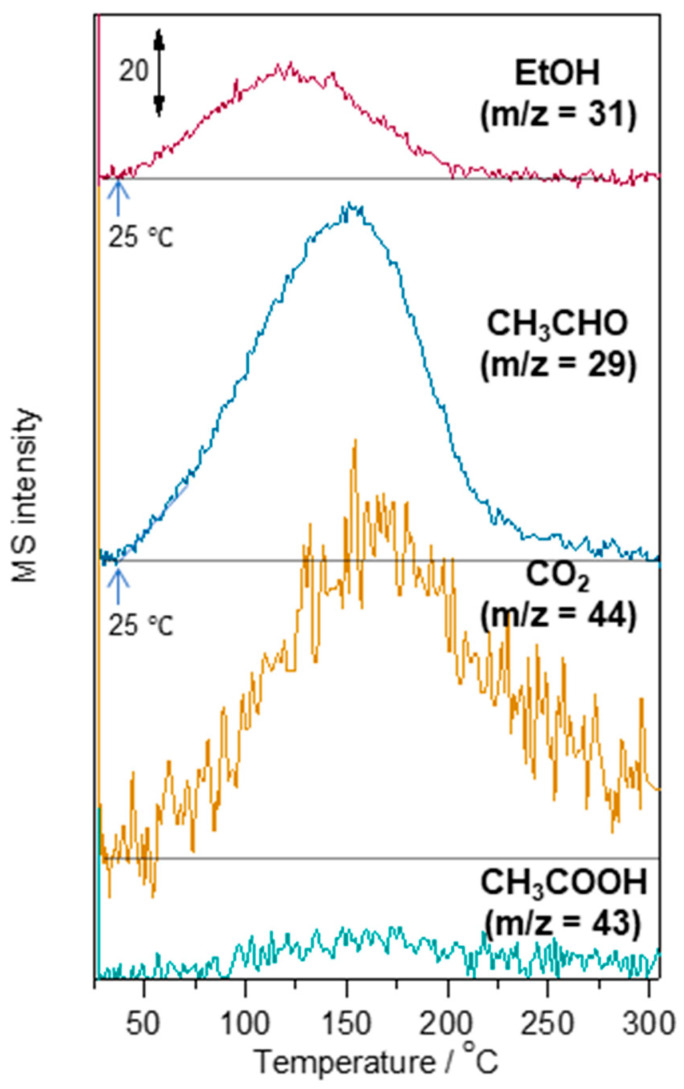
Temperature programmed desorption (TPD) profiles of adsorbed ethanol and by-products on Au-WO_3_.

To confirm the catalysis of ethanol and the formation of by-products on Au-WO_3_, TPD studies were performed. Figure 9 shows the TPD profiles of ethanol, acetaldehyde, CO_2_, and acetic acid for Au-WO_3_. The mass intensity for ethanol gradually increased at 25 °C and peaked at 120 °C for Au-WO_3_, indicating that ethanol desorption started at 25 °C. The acetaldehyde and CO_2_ desorption also started at 25 °C. The results suggest that ethanol combustion occurred at room temperature and acetaldehyde was one of the by-products of ethanol combustion. As revealed above, increasing the sensing layer thickness and the operation temperature decreased the sensor response. The TPD results support the hypothesis that the ethanol diffusion to the TPB was retarded by the heterogeneous catalytic combustion, which can be accelerated by heating the sensor at a higher temperature and increasing the sensing layer thickness. It should be noted that the desorption peak temperature for ethanol was lower than those for acetaldehyde, acetic acid, and CO_2_, suggesting that those molecules more strongly bind to Au-WO_3_ than ethanol. Thus, the slow recovery of the sensor response observed should be due to the slow desorption of CO_2_ and acetaldehyde from the sensor.

## 4. Conclusions

Graphene oxide (GO) was synthesized by a modified Tour’s method using expanded graphite as a starting material. Self-standing GO membranes intercalated with Ce ions were fabricated by simple vacuum filtration using a GO suspension containing Ce(SO_4_)_2_. The addition of Ce in GO membranes enhanced their proton conductivity as well as stability. The gas sensor device was fabricated using the Ce-doped GO membrane, Au-WO_3_/rGO sensing electrode, and Pt-C counter electrode. The SEM, TEM, and Raman results revealed that Au nanoparticles (10–80 nm) were deposited on WO_3_ particles (6–14 µm) that were wrapped by rGO nanosheets. The sensor with the Au-WO_3_/rGO sensing electrode showed good ΔV responses to ethanol (10–40 ppm) in the air at room temperature to 60 °C. The largest sensor response was observed at room temperature. Furthermore, the sensor showed low responses to H_2_ and CO, demonstrating a good selectivity to ethanol. The ΔV response linearly changed as a function of the logarithm of ethanol concentration on the basis of the mixed-potential theory. The thickness of the sensing electrode significantly affected the sensor performance. Decreasing the thickness from 177 to 34 µm much improved the sensor response, enabling the detection of ethanol at ppb concentrations in air at room temperature. The efficient combustion of ethanol in the thick sensing layer is responsible for the decreased sensor response, as revealed by TPD studies. The major drawback of the present sensor is its slow response and recovery. Thus, further increasing the electrochemical reaction rate through electrode materials development is necessary.

## Supplementary Materials

The following supporting information can be downloaded at: https://www.mdpi.com/article/10.3390/s22093194/s1, Figure S1: Thermogravimetric analysis; Figure S2: N_2_ adsorption isotherm and pore size distribution; Table S1: Total pore volume, mean pore size, and specific surface area.
