# Supplementary material for: Electrochemical Detection of Ethanol in Air Using Graphene Oxide Nanosheets Combined with Au-WO3
Source: Sensors (Basel). 2022 Apr 21;22(9):3194. doi: 10.3390/s22093194 (PMC9105121; doi:10.3390/s22093194)
Supplement: Supplementary file 1 [file sensors-22-03194-s001.zip › sensors-1678287-supplementary.pdf]

Supplementary Material

Electrochemical Detection of Ethanol in Air Using Graphene Oxide Nanosheets Combined with Au-WO_3

Aynul Sakinah Ahmad Fauzi [1], Nur Laila Hamidah [2], Shota Kitamura [1], Taiga Kodama [1], Kosuke Sonda [1], Ghina Kifayah Putri [1], Takeshi Shinkai [1], Muhammad Sohail Ahmad [3], Yusuke Inomata [4,5], Armando T. Quitain [5,6] and Tetsuya Kida [3,4,5,*]

1 Department of Material Science and Applied Chemistry, Graduate School of Science and Technology,
Kumamoto University, Kumamoto 860-8555, Japan; aynulsakinahaf@student.usm.my (A.S.A.F.); 207d5214@st.kumamoto-u.ac.jp (S.K.); 210d8811@st.kumamoto-u.ac.jp (T.K.); 212d8815@st.kumamoto-u.ac.jp (K.S.); 212d8829@st.kumamoto-u.ac.jp (G.K.P.); 211d9402@st.kumamoto-u.ac.jp (T.S.)

2 Department of Engineering Physics, Institut Teknologi Sepuluh Nopember (ITS), Surabaya 60111, Indonesia; nurlaila@its.ac.id

3 Institute of Industrial Nanomaterials, Kumamoto University, Kumamoto 860-8555, Japan;
sohail@kumamoto-u.ac.jp

4 Division of Materials Science, Faculty of Advanced Science and Technology, Kumamoto University,
Kumamoto 860-8555, Japan; inomata@kumamoto-u.ac.jp

5 International Research Organization for Advanced Science and Technology (IROAST), Kumamoto University, Kumamoto 860-8555, Japan; quitain@kumamoto-u.ac.jp

6 Center for International Education, Kumamoto University, Kumamoto 860-8555, Japan

* Correspondence: tetsuya@kumamoto-u.ac.jp

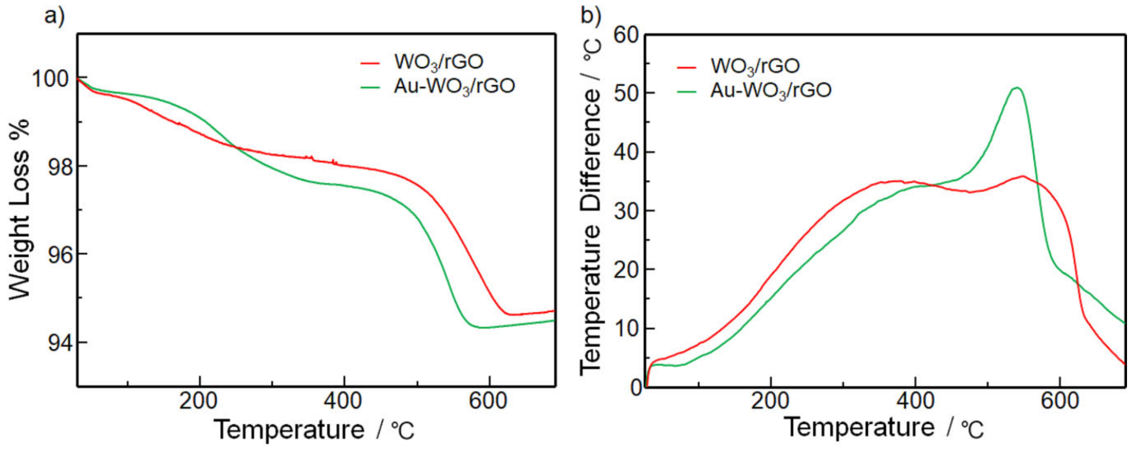

Figure S1. a) Thermogravimetric analysis (TGA) and b) differential thermal analysis (DTA) curves of WO_3/rGO and Au-WO_3/rGO in N_2 atmosphere.

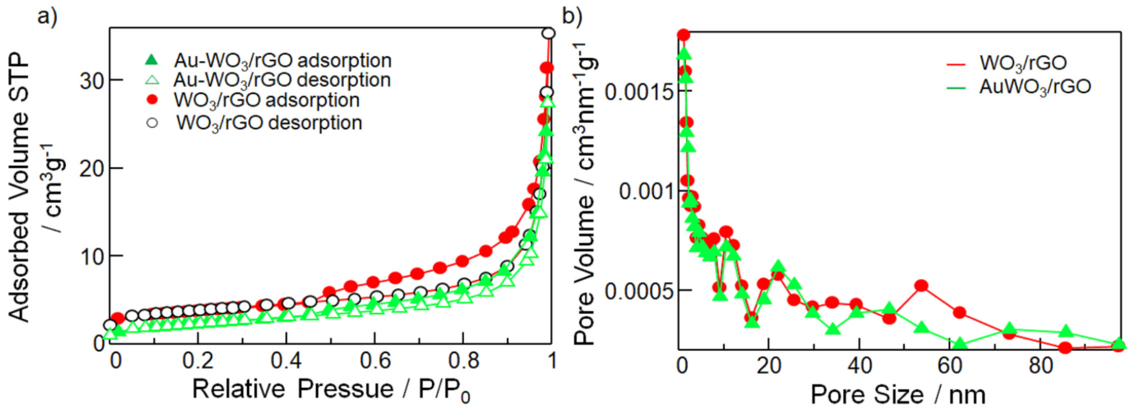

Figure S2. a) N_2 adsorption isotherm and b) pore size distribution of WO_3/rGO and Au-WO_3/rGO.

Table S1. Total pore volume, mean pore size and specific surface area of WO_3/rGO and Au-WO_3/rGO

	Total pore volume (cm^3/g)	Pore size (nm)	Specific surface area (m^2/g)
WO_3/rGO	0.045	13.7	2.38
**Au-WO_3/rGO**	0.040	19.9	8.22
